# Fibroblast activation protein-targeted radionuclide therapy: background, opportunities, and challenges of first (pre)clinical studies

**DOI:** 10.1007/s00259-023-06144-0

**Published:** 2023-02-23

**Authors:** Bastiaan M. Privé, Mohamed A. Boussihmad, Bart Timmermans, Willemijn A. van Gemert, Steffie M. B. Peters, Yvonne H. W. Derks, Sanne A. M. van Lith, Niven Mehra, James Nagarajah, Sandra Heskamp, Harm Westdorp

**Affiliations:** 1 Department of Radiology and Nuclear Medicine, PO Box 9101, Radboudumc, 6500 HB Nijmegen, The Netherlands; 2 Department of Radiation Oncology, Erasmus MC, Rotterdam, The Netherlands; 3 Department of Medical Oncology, Radboudumc, Nijmegen, The Netherlands

**Keywords:** Fibroblast activation protein, Cancer-associated fibroblasts, Radionuclide therapy, Lutetium-177, Yttrium-90

## Abstract

**Introduction:**

Fibroblast activation protein (FAP) is highly overexpressed in stromal tissue of various cancers. While FAP has been recognized as a potential diagnostic or therapeutic cancer target for decades, the surge of radiolabeled FAP-targeting molecules has the potential to revolutionize its perspective. It is presently hypothesized that FAP targeted radioligand therapy (TRT) may become a novel treatment for various types of cancer. To date, several preclinical and case series have been reported on FAP TRT using varying compounds and showing effective and tolerant results in advanced cancer patients. Here, we review the current (pre)clinical data on FAP TRT and discuss its perspective towards broader clinical implementation.

**Methods:**

A PubMed search was performed to identify all FAP tracers used for TRT. Both preclinical and clinical studies were included if they reported on dosimetry, treatment response or adverse events. The last search was performed on July 22 2022. In addition, a database search was performed on clinical trial registries (date 15^th^ of July 2022) to search for prospective trials on FAP TRT.

**Results:**

In total, 35 papers were identified that were related to FAP TRT. This resulted in the inclusion of the following tracers for review: FAPI-04, FAPI-46, FAP-2286, SA.FAP, ND-bisFAPI, PNT6555, TEFAPI-06/07, FAPI-C12/C16, and FSDD.

**Conclusion:**

To date, data was reported on more than 100 patients that were treated with different FAP targeted radionuclide therapies such as [^177^Lu]Lu-FAPI-04, [^90^Y]Y-FAPI-46, [^177^Lu]Lu-FAP-2286, [^177^Lu]Lu-DOTA.SA.FAPI and [^177^Lu]Lu-DOTAGA.(SA.FAPi)_2_. In these studies, FAP targeted radionuclide therapy has resulted in objective responses in difficult to treat end stage cancer patients with manageable adverse events. Although no prospective data is yet available, these early data encourages further research.

**Supplementary Information:**

The online version contains supplementary material available at 10.1007/s00259-023-06144-0.

## Introduction

According to Global Cancer Statistics, there were roughly 19.3 million new cancer diagnoses and 10.0 million cancer deaths globally in 2020 [1]. Cancers develop in complex environments composed of tumor cells and the surrounding stroma. As early as 1889, the “seed and soil” idea highlighted the interdependent relevance of both elements [2]. The majority of diagnostic and therapeutic approaches have focused on tumor cells. However, the tumor microenvironment (TME) is receiving incremental attention.

**Fig. 1:**
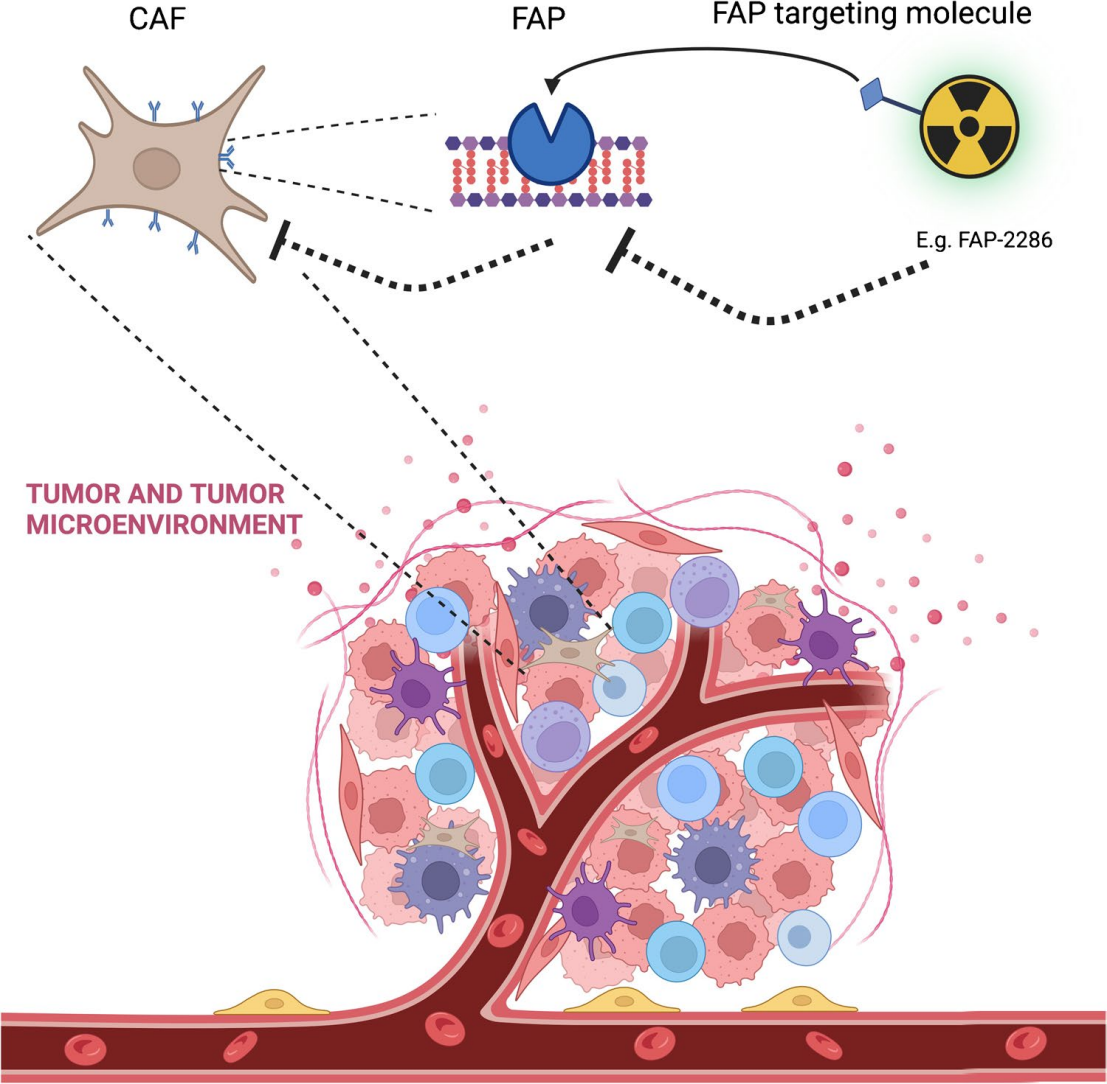
Cancer-associated fibroblast in the tumor microenvironment. CAF, cancer-associated fibroblasts; FAP, fibroblast activation protein

The TME consists of immune cells, vasculature, extracellular matrix, and cancer-associated fibroblasts (CAF). Treatment failure can occur as a result of the development of an immunosuppressive TME that shields tumor cells from therapeutic agents. In solid tumors, CAFs are one of the TMEs most prevalent components [3]. However, CAFs are heterogeneous cells, and various conditions have been shown to result in both tumor-promoting and tumor-suppressive activities [4]. They are able to remodel the ECM structure, thereby contributing to tumor initiation, neovascularization, and metastasis. This can either act as a physical barrier to prevent the infiltration of immune cells or as a structural scaffold for intercellular interaction between tumor cells and non-tumor cells in the TME [5]. Adversely, CAFs can release a variety of chemokines and cytokines, including interleukin-6 (IL-6), CC-chemokine ligand 2, and transforming growth factor (TGF), in order to attract inhibitory immune cell subsets in the tumor stroma, hence promoting immune evasion [6]. Platelet-derived growth factor receptor-β (PDGFR-β), α-smooth muscle actin (α-SMA), and fibroblast activation protein (FAP) are only a few of the biological markers found in CAFs. FAP is a type II integral membrane glycoprotein from the serine protease family that has a role in fibrogenesis and ECM remodeling. Because of the wide distribution of FAP in numerous cancer types (e.g., sarcoma, prostate cancer, breast cancer, lung cancer, pancreatic cancer, head and neck cancer, colorectal cancer) the question arises whether FAP can be a novel therapeutic target in cancer (Fig. 1).

Despite the literature’s inconsistent findings about the prognostic significance of FAP in malignancies, high expression of FAP has been demonstrated to be an independent poor prognostic predictor for outcomes in lung cancer, hepatocellular carcinoma, and colon cancer [7, 8]. FAP overexpression has been shown to enhance tumor development in a mouse model which may afterwards be decreased by anti-FAP antibodies [9]. However, sibrotuzumab (a humanized version of the mouse anti-FAP antibody) was ineffective as a therapy for metastatic colorectal cancer in an early phase II study [10]. Yet, the field of nuclear medicine has recently seen an increase in interest of radiolabeled small molecules that target FAP. It has been hypothesized that positron emission tomography (PET) of radiolabeled FAP inhibitors (FAPI) with short-living positron emitters (e.g., ^68^Ga/^18^F) may replace ^18^F-FDG in several major tumor types (e.g., breast cancer, pancreatic cancer, sarcoma) [3, 11]. Moreover, due to the high tumor uptake of FAP tracers and low expression of FAP in healthy tissues, FAP is deemed as a promising target for FAP TRT. As response to TRT is a result of the cytotoxic effect of radiation and not to a direct inhibitory effect of a FAP-binding molecule, the earlier reports on anti-FAP antibodies (e.g., sibrotuzumab) does not apply using this approach. While the FAP-targeting agent ensures uptake in the tumor stroma, the cross-fire effect of the radiolabeled (alpha or beta emitting) radionuclides deliver tumoricidal doses to the ECM including the cancer cells. Currently, several clinical trials are investigating ^177^Lu, ^90^Y, and ^225^Ac-labeled FAP-targeting tracers (e.g., NCT04939610, NCT04849247, ACTRN12621000935831), and several small case series have been published to date. Therefore, we aimed to review the presently reported results on therapeutic FAP ligands for TRT and provide an overview of the active studies. Finally, potential opportunities and challenges are discussed.

## Method

### Search strategy

The selection of FAP ligand types for this review was based on a combination of electronic library searches. First, the different FAP ligands used for TRT were analyzed. Second, literature was searched by universal PubMed searches (see Supplementary Table 1) for the FAP ligands. This resulted in the inclusion of FAPI-04, FAPI-46, FAP-2286, SA.FAP, ND-bisFAPI, PNT6555, TEFAPI-06/07, FAPI-C12/C16, and FSDD.

For each FAP-targeting molecule, the PubMed results were screened for preclinical and clinical studies on FAP TRT. Both preclinical and clinical studies and conference abstracts were included. The last search was performed on the 22 of July 2022.

For preclinical studies, data was reported on the percentage of injected dose per gram (%ID/g), dosimetry, and treatment response. Retrospective as well as prospective data of clinical studies were included if they reported on either dosimetry, treatment response, or adverse events. FAP imaging studies were excluded from this report.

A database search was performed on clinical trial registries such as clinicaltrials.gov (date 15 of July 2022) to search for active prospective trials using the terms: fibroblast activation protein, cancer-associated fibroblasts, FAP, and FAPI.

## Results

### mAbF19

The first FAP TRT study was published in 1994. In this study, Welt et al. described the toxicity, imaging and biodistribution characteristics of ^131^I (beta decay, t_1/2_ 8 days) labeled with the monoclonal antibody F19 ([^131^I]I-mAbF19) [12]. Seventeen patients with hepatic metastases from colorectal cancer were given [^131^I]I-mAbF19 intravenously. Respectively, seven, four, and six patients received 0.2, 2, or 8 mg/m^2^ of mAbF19 labeled with ~ 370 MBq ^131^I, approximately 7 days prior to surgery. Visualization of [^131^I]I-mAbF19 accumulation was made possible by the γ-decay of ^131^I. Fifteen of the 17 patients showed selective uptake of [^131^I]I-mAbF19 in metastases, with a 21:1 tumor to liver ratio. Administration of [^131^I]I-mAbF19 was not linked to any toxicities. At the time of surgery, between 0.001 and 0.016% of the administered [^131^I]I-mAbF19 dose per gram tumor (%ID/g tumor) were detected in the metastases. The authors suggested potential diagnostic and therapeutic application of humanized [^131^I]I-mAbF19. Based on the results with the murine mAb F19, and to address problems of immune responses to murine antibodies, a few years later, a humanized version of the mAb F19 (sibrotuzumab) was developed. In a phase I dose escalation study of [^131^I]I-sibrotuzumab study, 26 patients (*n* = 20 colorectal cancer and *n* = 6 with non-small lung cancer) received either 5 mg/m^2^, 10 mg/m^2^, 25 mg/m^2^, or 50 mg/m^2^ sibrotuzumab labeled with 296–370 MBq ^131^I. A total of 218 infusions of sibrotuzumab were administered during the first 12 weeks of the study, with 24 patients being evaluable. Three patients were excluded from study because of symptoms of clinical immune responses to the humanized antibody. Unfortunately, both studies on FAP TRT failed to show significant efficacy with no objective tumor responses observed [12, 13]. Therefore, FAP TRT was rested, until the last few years when novel small-molecules targeting FAP were developed.

### FAPI-04

#### Preclinical studies

The first study on FAP inhibitors (FAPI) was published in 2018 by Lindner and colleagues. In this study, BALB/c *nu/nu* mice were inoculated with HT-1080 cells transfected with FAP. When the tumor had grown to approximately 1 cm^3^, several different [^177^Lu]Lu-labeled FAPIs were injected via the tail vein. In this study, FAPI-04 was the best candidate for TRT as it showed high tumor accumulation, slower excretion, and no significant increase in background activity. While [^177^Lu]Lu-FAPI-13 showed higher tumor uptake leading to higher doses, the retention of the tracer in the blood was also higher. It was hypothesized that this may lead to higher bone marrow toxicity during treatment. Therefore, FAPI-04 was chosen as the preferred theranostic tracer despite having a rather short tumor retention time. The authors suggest using FAPI-04 with a radionuclide that follows this shorter retention time (e.g., ^153^SM, beta decay, and t_1/2_ of 1.92 days or ^90^Y, beta decay, and t_1/2_ of 2.66 days).

In 2020, Watabe et al. evaluated FAPI-04 in a murine tumor model [14]. Mice with human pancreatic cancer xenografts (PANC-1 or MIA PaCa-2) received 7.21 ± 0.46 MBq [^64^Cu]Cu-FAPI-04 (t_1/2_ 12.7 h) to enable PET imaging. For TRT, PANC-1 xenografted mice received 34 KBq of [^225^Ac]Ac-FAPI-04. When compared to untreated control mice, [^225^Ac]Ac-FAPI-04 injection significantly reduced tumor growth without significantly altering body weight. Following these results, the authors postulated that [^225^Ac]Ac-FAPI-04 showed potential for treatment of pancreatic cancer. In 2021, Ma et al. described a preclinical study of FAPI-04 [15]. The authors hypothesize that ^211^At may be best suited for FAPI-04 TRT due to the alpha decay and the short T_1/2_ of 7.2 h compared to ^90^Y, ^177^Lu (beta decay, t_1/2_ 6.7 days), ^225^Ac (alpha decay, t_1/2_ 10 days), and ^188^Re (beta decay, T_1/2_ 16.9 hours). However, due to limited availability of ^211^At, they used ^131^I as it has similar chemical characteristics to ^211^At. In this work, FAPI-02 and FAPI-04 precursors were synthesized for ^211^At and ^131^I labeling. [^131^I]I-FAPI-04 produced good tumor accumulation and good tumor-to-organ ratios, which were shown by SPECT/CT imaging. Additionally, intratumoral injection of [^131^I]I-FAPI-04 effectively reduced tumor development in U87MG xenograft mice side effects. In January 2022, Ma et al. described the in vitro and in vivo therapeutic effect of [^211^At]At-FAPI-04 in a glioma tumor model [16]. Four-week-old BALB/c-nude mice were inoculated with U87MG and MCF-7 cells. [^211^At]At-FAPI-04 (0.3 MBq) was administrated to U87MG xenografts via intravenous or intratumoral injection when the tumor volume reached ~ 200 mm^3^. [^211^At]At-FAPI-04 clearly inhibited tumor growth and increased median survival in a dose-dependent manner without significantly harming healthy organs. [^211^At]At-FAPI-04 therapy was similarly accompanied with enhanced apoptosis and decreased proliferation. All of these findings implied that [^211^At]At-FAPI-04 may be a successful approach for TRT with FAPI-04. No clinical trial using [^211^At]At-FAPI-04 has been reported to date.

#### Clinical data

The study of Lindner and colleagues in 2018 presented a patient with metastasized breast cancer who received ~ 2.9 GBq [^90^Y]Y-FAPI-04. Imaging with [^68^Ga]Ga-FAPI-04 and [^90^Y]Y-FAPI-04 revealed high tracer uptake in metastases. After treatment with [^90^Y]Y-FAPI-04, the patient reported significantly less pain. Furthermore, no side effects were observed, especially with respect to the bone marrow [17]. The authors anticipated that higher activities can be given resulting in better tumoricidal effects.

In 2021, Kuyumcu et al. published a dosimetry study where a low dose of [^177^Lu]Lu-FAPI-04 (267.5 ± 8.6 MBq) was given to four patients with metastatic advanced-stage cancer (breast cancer, thymic carcinoma, thyroid cancer, and ovarian cancer) [18]. Data acquisition was obtained using whole-body SPECT/CT imaging, and blood samples were collected for bone marrow dosimetry. With [^177^Lu]Lu-FAPI-04, the mean absorbed dose to healthy organs at risk was low: 0.25 ± 0.16 mGy/MBq (range: 0.11–0.47 mGy/MBq), 0.11 ± 0.08 mGy/MBq (range: 0.06–0.22 mGy/MBq), and 0.04 ± 0.002 mGy/MBq (range: 0.04–0.046 mGy/MBq) for kidneys, liver, and bone marrow, respectively (Table 1). For bone, lymph node, liver, and soft tissue metastases, the mean absorbed dose was 0.62 ± 0.55 mGy/MBq, 0.38 ± 0.22 mGy/MBq, 0.33 ± 0.21 mGy/MBq, and 0.37 ± 0.29 mGy/MBq, respectively. Therefore, the dose to the organs at risk was lower compared to the dose to tumors. However, the authors concluded that employing different radioisotopes may be preferred due to the short tumor retention time of FAPI-04. Moreover, development of tracers with better tumor retention was warranted.

**Table 1:** Clinical outcomes of FAP targeted radionuclide therapy

Year	Author	Drug	Total GBq	N	Tumor	Response	Side effects	Tumor dosimetry (Gy/GBq)	Bone marrow dosimetry (Gy/GBq)	Kidney dosimetry (Gy/GBq)
2018	Linder et al. [17]	[^90^Y]Y-FAPI-04	2.9	1	Breast	Less pain	None	NR	NR	NR
2021	Kuyumcu et al. [18]	[^177^Lu]Lu-FAPI-04	0.27	4	Breast, thymus, thyroid, and ovarium	NR	NR	0.62	0.002	0.25
2020	Linder et al. [21]	[^90^Y]Y-FAPI-46	6	2	Ovarium and pancreas	NR	NR	NR	NR	NR
2021	Kratochwil et al. [22]	[^153^Sm]Sm/[^90^Y]Y-FAPI-46	20 + 8	1	Sarcoma	Stable disease	None	NR	NR	NR
2021	Rathke et al. [23]	[^90^Y]Y-FAPI-46	Up to 28.1	1	Colorectal and breast	Mixed response	None	NR	NR	NR
2021	Assadi et al. [24]	[^177^Lu]Lu-FAPI-46	1.85–4.44	18	Various (breast, colon, ovarium, etc.)	NR	1p grade I–III hematotoxicity	NR	NR	0.886
2022	Kaghazchi et al. [25]	[^177^Lu]Lu-FAPI-46	1.85	1	Pancreas	NR	None	NR	NR	NR
2022	Barashki et al. [26]	[^177^Lu]Lu-FAPI-46	7.4	1	Multiple endocrine neoplasia type 2A	Clinical improvement	None	NR	NR	NR
2022	Fu et al. [27]	[^177^Lu]Lu-FAPI-46	22.3	1	Radioiodine-refractory thyroid cancer	NR	None	NR	NR	NR
2022	Fu et al. [28]	[^177^Lu]Lu-FAPI-46	3.7	1	Nasopharyngeal	Mixed response	None	NR	NR	NR
2022	Ferdinandus et al. [29]	[^90^Y]Y-FAPI-46	3.8–7.8	10	Pancreas and sarcoma	NR	4p grade III hematotoxicity	1.28	0.04	0.52
2022	Fendler et al. [30]	[^90^Y]Y-FAPI-46	3.7–7.4	21	Sarcoma, pancreas, prostate, gastric	1p partial response, 7p stable disease	8p (38%) grade > III mostly hematotoxicity	2.81	0.04	0.53
2021	Baum et al. [33]	[^90^Y]Y-FAP-2286	5.8	11	Pancreas, breast, rectum, and ovarium	2p with stable disease, 3p pain relief	3p grade III, mostly hematotoxicity	3	0.05	1
2022	McConathy et al. [34]	[^177^Lu]-FAP-2286	3.7	3	Colorectal and peritoneal	NR	No grade III/IV	NR	NR	NR
2021	Ballal et al. [36]	[^177^Lu]Lu-DOTA.SA.FAPI	3.2	1	Breast	Less pain	None	1.48-3.46	NR	NR
2021	Ballal et al. [37]	[^177^Lu]Lu-DOTA.SA.FAPI	2.96	3	Breast	NR	NR	0.6	0.001	0.62
2021	Ballal et al. [37]	[^177^Lu]Lu-DOTAGA.(SA.FAPI)_2_	1.48	7	Thyroid, breast, and paraganglioma	NR	NR	6.7	0.02	0.37
2022	Ballal et al. [38]	[^177^Lu]Lu-DOTAGA.(SA.FAPI)_2_	8.2	15	Radioiodine-refractory differentiated thyroid cancer	4p with partial response, 3p with stable disease	No grade III/IV	10.8	0.3	0.026

*NR*, not reported; *P*, patients

### FAPI-46

#### Preclinical studies

In recent years, the group of the University of Heidelberg (Germany) studied several FAPI compounds in the search for a FAP tracer with the best properties for TRT. FAPI-46 proved to be most suitable as a theranostic agent due to a longer tumor retention (compared with FAPI-04) while having a similar uptake in healthy tissues [19].

The therapeutic response of [^177^Lu]Lu-FAPI-46 and [^225^Ac]Ac-FAPI-46 was evaluated in a pancreatic cancer model [20]. Eighteen PANC-1 xenograft mice received 3 MBq (*n* = 6), 10 MBq (*n* = 6), or 30 MBq (*n* = 6) [^177^Lu]Lu-FAPI-46, and 11 PANC-1 xenograft mice were injected with 3 kBq (*n* = 3), 10 kBq (*n* = 2), or 30 kBq (*n* = 6) [^225^Ac]Ac-FAPI-46. Both [^177^Lu]Lu-FAPI-46 and [^225^Ac]Ac-FAPI-46 demonstrated quick renal excretion and resulted in a tumor uptake of ~ 0.3 %ID/g at 3 h and ~ 0.1 %ID/g at 24 h. Kidney uptake was approximately 1–2 %ID/g at 3 h and 0.2–0.5 %ID/g at 24 h for both [^177^Lu]Lu-FAPI-46 and [^225^Ac]Ac-FAPI-46. Both [^177^Lu]Lu-FAPI-46 and [^225^Ac]Ac-FAPI-46 showed tumor suppression along with a little reduction in body weight. The authors postulated that FAPI TRT may have a role in pancreatic cancer. However, radionuclides with shorter half-life seem more suitable for FAPI-46-based therapy.

#### Clinical data

The first clinical data in humans on FAPI-46 was published in 2020. This study reported on a patient with metastasized ovarian cancer and a patient with pancreatic cancer that both received 6 GBq [^90^Y]Y-FAPI-46 as a last-line treatment. Significant tumor uptake was seen, with low uptake in healthy organs. No follow-up data was reported of these patients [21]. Another study showed a patient with metastatic fibrous spindle cell soft tissue sarcoma that was treated with 20 GBq [^153^Sm]Sm-FAPI-46 and 8 GBq [^90^Y]Y-FAPI-46 (in an unreported amount of cycles) [22]. The patient received FAPI TRT following all standard of care treatments. FAPI TRT was well tolerated and resulted in stable disease for 8 months. Later, Rahtke and colleagues described a patient with metastatic breast and colorectal cancer, who progressed after several lines of chemotherapy [23]. Both colorectal and breast cancer metastases showed tumor targeting on [^68^Ga]Ga-FAPI-04 PET/CT. The patient received 3 cycles with a total of 28.1 GBq [^90^Y]Y-FAPI-46. Radioligand treatment had a mixed effect with the peritoneal metastases disappearing, but only a little success in treating the breast cancer metastases (despite similar tracer uptake on the FAPI-PET/CT). The overall survival (OS) of this patient was 11 months. At the end of 2021, Assadi et al. demonstrated the therapeutic potential and the feasibility of [^177^Lu]Lu-FAPI-46 in patients with advanced, non-operable tumors or tumors that were refractory to conventional therapies [24]. Eighteen patients (ovarian cancer (*n* = 2), sarcoma (*n* = 1), colon cancer (*n* = 3), breast cancer (*n* = 5), pancreatic cancer (*n* = 2), prostate cancer (*n* = 1), cervical cancer (*n* = 1), round-cell tumor (*n* = 1), lung cancer (*n* = 1), and anaplastic thyroid cancer (*n* = 1)) were treated with doses of 1.85–4.44 GBq [^177^Lu]Lu-FAPI-46 per cycle. Ten patients received 2 or more cycles of [^177^Lu]Lu-FAPI-46. Therapy was performed in a *compassionate use* setting. Nearly all patients tolerated the treatment well. However, there was one sarcoma patient with grade 3 anemia, grade 1 thrombocytopenia, and grade 1 leukopenia. Another patient complained of pain increment following treatment. No clear progression-free or OS data could be drawn from this study due to its retrospective design. The median absorbed dose to the kidneys was 0.886 (0.076–1.39) mGy/MBq. The authors did not report on tumor or bone marrow dosimetry. All in all, the authors postulated that [^177^Lu]Lu-FAPI-46 seems feasible and safe.

In the beginning of 2022, four case reports were published. The first is a patient with end-stage metastatic pancreatic ductal adenocarcinoma with no standard of care options available. The patient was treated with 1.85 GBq [^177^Lu]Lu-FAPI-46 [25]. [^177^Lu]Lu-FAPI-46 showed gradual washout of the tumor lesions within 6 days. The OS in this patient was 45 days. The authors suggested that FAPI-46 may not be the ideal tracer for theranostic applications. The second case report suggested otherwise. Here, TRT with [^177^Lu]Lu-FAPI-46 was described in a patient with multiple endocrine neoplasia type 2A syndrome [26]. This patient was also out of conventional options and received 7.4GBq [^177^Lu]Lu-FAPI-46. Following treatment, the patient reported clinical improvement. No grade > 3 AEs were observed. The third case report described a radioiodine-refractory thyroid cancer patient with no conventional treatment options. The patient was treated with four cycles of in total 22.3 GBq [^177^Lu]Lu-FAPI-46 [27]. Uptake was observed 72-h post injection on whole-body scintigraphy. Follow-up exams showed stable disease per Response Evaluation Criteria in Solid Tumors (RECIST) 1.1. No therapy-related adverse symptoms were reported. The authors claimed that [^177^Lu]Lu-FAPI-46 may be useful in the treatment of advanced radioiodine-refractory thyroid cancer. Lastly, a case report showed the use of 3.7 GBy [^177^Lu]Lu-FAPI-46 in a 25-year-old patient with undifferentiated nasopharyngeal carcinoma [28]. No therapy-related AEs were observed. Eight weeks after TRT, imaging demonstrated a mixed response. The patient had an OS of 3 months.

More recently, a larger case series was published using [^90^Y]Y-FAPI-46 and reported on its safety, dosimetry, and viability [29]. Between June 2020 and March 2021, nine patients underwent treatment for pancreatic cancer (*n* = 3) or metastatic soft-tissue or bone sarcoma (*n* = 6). All patients received a median activity of 3.8 GBq for their first cycle, and three patients received 2–3 cycles with a median of 7.4 GBq per cycle. The median follow-up was 44 days (IQR 36–84 days). Five patients died during follow-up. All five deaths were considered to be due to tumor progression and not related to [^90^Y]Y-FAPI-46. Overall, FAP TRT with [^90^Y]Y-FAPI-46 was well tolerated and showed few AEs. However, four patients developed grade 3 bone marrow toxicity probably related to TRT. Although there were indications of a tumor response, the follow-up was too short for proper response evaluation. Median bone marrow and renal absorbed dose were 0.04 Gy/GBq (IQR, 0.03–0.06 Gy/GBq) and 0.52 Gy/GBq (IQR, 0.41–0.65 Gy/GBq) per cycle, respectively. They suggested that more cycles of [^90^Y]Y-FAPI-46 were feasible given the low radiation doses to organs at risk. Median absorbed dose for tumor lesions after the first cycle was 1.28 Gy/GBq (IQR, 0.83–1.71 Gy/GBq) per cycle for target lesions. In July 2022, the third and latest published study using [^90^Y]Y-FAPI-46 was reported by Fendler et al. (which potentially included patients of the prior study) [30]. The effectiveness (following RECIST) and safety (following CTCAE 5.0) of [^90^Y]Y-FAPI-46 were reported in in 16, 3, 1, and 1 patient(s) with sarcoma, pancreatic, prostate, and gastric cancer. These 21 patients, received 47 cycles of 3.7–7.4GBq [^90^Y]Y-FAPI-46-RLT. Thirteen out of the 21 patients received two or more cycles. Eight (38%) patients experienced a grade 3 or 4 adverse event, which were mostly bone marrow toxicities. A response per RECIST was seen in approximately a third of the patients. There was 1 partial response and 7 stable diseases. Median progression-free survival (95% CI) was 3.4 (1.1–5.7) months. The median OS was significantly longer for patients that had a RECIST response (14.4 months vs. 6.6 months). The mean (standard deviation [±]) radiation dose was 0.53 ± 0.04, 0.04 ± 0.01, 2.81 ± 1.25, and 2.15 ± 0.67 Gy/GBq for kidney, bone marrow, tumor lesion with highest radioligand uptake, and lesion with second highest uptake. None of the organs at risk reached threshold doses (Table 1). The authors concluded that [^90^Y]Y-FAPI-46 was well tolerated and had organ radiation doses below critical range. More studies are awaited.

### FAP-2286

#### Preclinical trials

The first preclinical data of FAP-2286 was published in 2020 by Zboralski et al. [31]. Following intravenous injection, in vivo biodistribution in mice showed renal excretion of FAP-2286, little uptake in normal tissues, and fast absorption of ^111^In and [^177^Lu]Lu-labelled FAP-2286 (30 MBq/nmol or 60 MBq/nmol) in FAP-positive tumors that were sustained for 120 h. After a single intravenous dose, [^177^Lu]Lu-FAP-2286 had a high anti-tumor activity in HEK-293 xenografts that expressed the FAP receptor. Tumor growth inhibition was ~ 115% at day 14 post-treatment with no significant weight loss. According to the authors, FAP-2286 offered an attractive profile for TRT due to its good tumor uptake and retention in FAP-positive tumors. [^177^Lu]Lu-FAP-2286 treatment was also compared to FAPI-46 in biochemical and cellular tests, in vivo imaging, and effectiveness experiments [32]. High affinity was shown for both cell surface FAP expressed on fibroblasts and FAP recombinant protein by FAP-2286 and its metal complexes. In mice, biodistribution investigations revealed that [^111^In]In-FAP-2286 (30 MBq) and [^177^Lu]Lu-FAP-2286 (30 MBq) rapidly and persistently accumulated in FAP-positive tumors, with urinary excretion and little uptake in healthy tissues. High tumor-to-background signal ratio was observed from 1-h post injection onward (in tumor: 11.1 % ID/g). Accumulation of [^111^In]In-FAP-2286 was stably maintained in the HEK-FAP tumors with 9.1 %ID/g at 48 h after injection. The organs with the highest non-target uptake were the kidneys. However, tumor to kidney ratio improved over time, with a 7.5 ratio of tumor to kidney at 48 h. In the HEK tumors that expressed FAP as well as xenografts derived from a sarcoma patient, [^177^Lu]Lu-FAP-2286 showed anti-cancer efficacy (e.g., 111% and 113% of tumor growth inhibition following 30 and 60 MBq [^177^Lu]Lu-FAP-2286, respectively) without causing any significant weight loss. In addition, compared to [^177^Lu]Lu-FAPI-46, [^177^Lu]Lu-FAP-2286 showed longer tumor retention (up to 72 h) and better tumor control. In conclusion, [^177^Lu]Lu-FAP-2286 showed encouraging preclinical data warranting clinical translation.

#### Clinical trials

The first-in-human results of [^177^Lu]Lu-FAP-2286 reported toxicity, dosimetry, and feasibility of 5.8 ± 2.0 GBq (per cycle) [^177^Lu]Lu-FAP-2286 in 11 patients with advanced pancreatic adenocarcinoma (*n* = 5), breast cancer (*n* = 4), rectal cancer (*n* = 1), and ovarian cancer (*n* = 1) [33]. One patient received a single cycle, 9 patients received 2 cycles, and 1 patient received 3 cycles. [^177^Lu]Lu-FAP-2286 showed tolerable side effects and prolonged tumor retention (up to 10 days). Three patients reported pain relief following treatment. According to RECIST 1.1, two patients had a stable disease, whereas the other nine had disease progression. No grade 4 adverse events were observed. Grade 3 events occurred in 3 patients: one developed pancytopenia, one leukopenia, and one pain increment. The mean absorbed doses were kidneys, 1.0 ± 0.6 Gy/GBq (range, 0.4–2.0 Gy/GBq); red marrow, 0.05 ± 0.02 Gy/GBq (range, 0.03–0.09 Gy/GBq); and bone metastases, 3.0 ± 2.7 Gy/GBq (range, 0.5–10.6 Gy/GBq) (Table 1). A clinical trial is currently recruiting patients to test [^177^Lu]Lu-FAP-2286 in a prospective setting.

At the 2022 conference of the Society of Nuclear Medicine, preliminary results of the LUMIERE trial (NCT04939610) were discussed. The LuMIERE study a prospective phase I/II study in patients with advanced or metastatic solid tumors evaluating the safety, dosimetry, pharmacokinetics, and preliminary anti-tumor activity of [^177^Lu]Lu-FAP-2286 [34]. The study started in 2021 and is estimated to be completed in 2026. At time of abstract submission, a total of 3 patients got treated with [^177^Lu]Lu-FAP-2286 (3.7 GBq, one patient received 3 cycli). None of the 3 patients reported any grade 3/4 AEs or clinically significant laboratory abnormalities. At a meeting later that year (European Association of Nuclear Medicine 2022), it was reported that 11 patients yet underwent [^177^Lu]Lu-FAP-2286 (3.7-7.4 GBq) with one patient having a partial response and one stable disease. The final results of this clinical trial are awaited.

### SA.FAPI

#### Preclinical data

In 2020, Moon et al. presented the first data on DOTA.SA.FAPi. This tracer was labelled with ^68^Ga and ^177^Lu with high radiochemical yield (> 97%) [35]. First proof-of-principle in vivo PET imaging animal studies of the [^68^Ga]Ga-DOTA.SA.FAPi precursor in a HT-29 human colorectal cancer xenograft mouse model indicated promising results with high accumulation in tumor and low background signal. Ex vivo biodistribution showed highest uptake in tumor (5.2 %ID/g) at 60-min post injection with overall low uptake in healthy tissues. However, [^68^Ga]Ga-DOTA.SA.FAPi also showed higher uptake in bone and small intestines. No in vivo results of [^177^Lu]Lu-DOTA.SA.FAPi were presented.

#### Clinical data

In September 2019, a 31-year-old woman was found to have advanced metastatic breast cancer (ER-PR-HER2neu+) [36]. On a *compassionate use* basis, the patient was given a single cycle of 3.2 GBq [^177^Lu]Lu-DOTA.SA.FAPI. In accordance with [^68^Ga]Ga-DOTA.SA.FAPI PET/CT scans, physiological radiotracer uptake of [^177^Lu]Lu-DOTA.SA.FAPI was seen in the liver, kidneys, pancreas, and background muscle uptake. Intense radiotracer accumulation was observed in all tumor lesions. After finishing the therapy, the patient observed clinical benefit by having less severe headache. No adverse treatment-related events were noted. The dose to the main tumor and the brain metastasis was approximately 1.48 mGy/MBq and 3.46 mGy/MBq, respectively. The next study of SA.FAPI reported on a total of ten patients (four with breast cancer, five patients with thyroid cancer, and one with paraganglioma). Three patients received 2.96 GBq [^177^Lu]Lu-DOTA.SA.FAPI, and seven patients were treated with 1.48 GBq [^177^Lu]Lu-DOTAGA.(SA.FAPI)_2_ [37]. No proper treatment response data was presented in this study. However, patients that received [^177^Lu]Lu-DOTAGA.(SA.FAPi)_2_ completed more cycles and had a longer survival compared to patients that received [^177^Lu]Lu-DOTA.SA.FAPi. The investigators found that [^177^Lu]Lu-DOTAGA.(SA.FAPI)_2_ had higher tumor-absorbed doses when compared to [^177^Lu]Lu-DOTA.SA.FAPI. The median absorbed doses to the lesions were 0.603 (IQR: 0.230–1.810) Gy/GBq and 6.70 (IQR: 3.40–49) Gy/GBq dose per cycle in the [^177^Lu]Lu-DOTA.SA.FAPi, and [^177^Lu]Lu-DOTAGA.(SA.FAPi)_2_ groups, respectively. The mean dose to the kidneys were 0.618 ± 0.015 Gy/GBq for [^177^Lu]Lu-DOTA.SA.FAPi, while the mean dose with [^177^Lu]Lu-DOTAGA.(SA.FAPi)_2_ was 0.374 ± 0.26 Gy/GBq. The dose to the bone marrow was 0.001 ± 0.0003 Gy/GBq vs. 0.0173 ± 0.0182 Gy/GBq with [^177^Lu]Lu-DOTA.SA.FAPi or [^177^Lu]Lu-DOTAGA.(SA.FAPi)_2,_ respectively. Moreover, both tracers had a higher uptake in colon of 0.43–0.47 Gy/GBq or 1.16–2.87 Gy/GBq using [^177^Lu]Lu-DOTA.SA.FAPi or [^177^Lu]Lu-DOTAGA.(SA.FAPi)_2_, respectively. All in all, this first dosimetry study demonstrated significantly tumor-absorbed doses with [^177^Lu]Lu-DOTA.SA.FAPi and [^177^Lu]Lu-DOTAGA.(SA.FAPI)_2_. The authors suggested that [^177^Lu]Lu-DOTAGA.(SA.FAPI)_2_ was preferred over [^177^Lu]Lu-DOTA.SA.FAPi.

Another study was published on [^177^Lu]Lu-DOTAGA.(SA.FAPI)_2_ in radioiodine-refractory differentiated thyroid cancer [38]. Forty-five cycles of [^177^Lu]Lu-DOTAGA.(SA.FAPI)_2_ were administered in 15 patients, with a median cumulative administered dose of 8.2 ± 2.7 GBq. Four patients had a partial response, and three patients showed stable disease. None showed grade III/IV toxicity. The median absorbed doses to tumor lesions, kidneys, bone marrow, and colon were 10.8 (IQR: 4.16 to 89.7), 0.3 ± 0.3 and 0.026 ± 0.02, 0.04 ± 1.97 mSv/MBq, respectively. These preliminary findings indicate that [^177^Lu]Lu-DOTAGA.(SA.FAPI)_2_ seemed safe, appears to be effective, and may provide a new therapeutic option for patients with aggressive radioiodine-refractory thyroid cancer who failed on all conventional therapy. A short while later, a case report emerged which described a 56-year-old man with aggressive medullary thyroid carcinoma, behaving clinically like anaplastic thyroid cancer. He was treated with one cycle of 1.65 GBq [^177^Lu]Lu-DOTAGA.(SA.FAPI)_2_. This resulted in a reduction of the tumor mass in the neck and improved quality of life [39]. Taken together, first results on [^177^Lu]Lu-DOTAGA.(SA.FAPI)2 are encouraging.

### ND-bisFAPI

There is one study on ND-bisFAPI [40]. The objective of this work was to create a bivalent FAP ligand that might be used for TRT as well as diagnostic PET imaging. In vitro studies of competitive binding to FAP, cellular internalization, and efflux characteristics were determined using FAP-positive A549-FAP cells and a murine tumor model. ND-bisFAPI demonstrated selective uptake, a high internalized proportion, and a delayed cellular efflux in A549-FAP cells. At 24, 72, 120, and 168 h, biodistribution analyses revealed that [^177^Lu]Lu-ND-bisFAPI had a greater tumor uptake than [^177^Lu]Lu-FAPI-04. A549-FAP tumors received four times more radiation from [^177^Lu]Lu-ND-bisFAPI than from [^177^Lu]Lu-FAPI-04. 37 MBq of [^177^Lu]Lu-ND-bisFAPI slowed tumor development considerably. However, the survival following [^177^Lu]Lu-ND-bisFAPI did not differ significantly from [^177^Lu]Lu-FAPI-04. All in all, ND-bisFAPI, which can be labeled with ^18^F and ^177^Lu, may be promising option for TRT. No clinical trial has been reported to date.

### PNT6555

PNT6555 is a novel FAP-targeting ligand. There is one conference abstract which described preclinical data of PNT6555 [41]. PNT6555 was labeled with ^68^Ga and ^177^Lu which showed potent activity in FAP inhibition assays using human, mouse, and rat sources of FAP. Direct organ analysis revealed little [^177^Lu]Lu-PNT6555 accumulation and retention in healthy tissues while revealing significant tumor retention up to 168 h (> 10 %ID/g). Importantly, [^177^Lu]Lu-PNT6555 showed rapid renal clearance. In a murine tumor model, a single dosage of [^177^Lu]Lu-PNT6555 or [^225^Ac]Ac-PNT6555 showed dose-dependent anti-tumor efficacy, with no apparent weight loss seen at any of the tested dosage levels. Clinical translation of [^68^Ga]Ga/[^177^Lu]Lu/[^225^Ac]Ac-PNT6555 is pending with its phase I–II trial recently starting recruiting (NCT05432193). This phase I trial will assess the safety and tolerability of [^68^Ga]Ga-PNT6555 and [^177^Lu]Lu-PNT6555 in patients with solid tumors that show FAP expression. Patients with FAP-avid disease on [^68^Ga]Ga-PNT6555 PET/CT will be eligible to receive up to 6 cycles of [^177^Lu]Lu-PNT6555.

### FAP tracers with albumin-binding moieties

#### EB-FAPI

FAP-targeted molecular imaging radiotracers have shown promising results; however, rapid clearance and inadequate tumor retention seem to be a recurring limitation. In order to overcome these limitations, FAPI tracers with albumin-binding moieties were developed. A group conjugated Evans Blue to the FAPI complex [42, 43]. Several EB-FAPI-Bn were synthesized, which were based on FAPI-02, and labeled with ^177^Lu. [^177^Lu]Lu-EB-FAPI-B1 showed high tumor accumulation in U87MG tumor bearing mice, with tumor retention up to 96-h post injection (12.42 ± 1.54 %ID/g). The kidney uptake of [^177^Lu]Lu-EB-FAPI-B1 was 16.38 ± 2.98 %ID/g 8-h post injection which then declined to 8.13 ± 1.36 %ID/g at 48-h post injection. Uptake in other healthy organs uptake was low.

Tumor growth inhibition was observed after administration of 7.4 MBq, 18.5 MBq, and 30 MBq [^177^Lu]Lu-EB-FAPI-B1. Slight weight loss was observed in each treatment groups but within normal range. There are two trials registered on clinicaltrials.gov using [^177^Lu]Lu-EB-FAPI. Both of these studies started in June 2022. The first study (NCT05400967) will be performed in patients with advanced cancer and will investigate dosimetry of [^177^Lu]Lu-EB-FAPI following an injection of 1.11 GBq. Moreover, they will also assess the safety and therapeutic response. In a second study NCT05410821, 20 radioactive iodine refractory thyroid cancer patients will be treated with up to 3 cycles of 1.11, 2.22, or 3.33 GBq [^177^Lu]Lu-DOTA-EB-FAPI. The aim of this phase II study is to assess safety, tolerability, and overall response rate.

#### TEFAPI-06 and TEFAPI-07

TEFAPI-06 and TEFAPI-07 are two albumin-binding FAPI tracers based on FAPI-04 than can be labeled with [44] ^68^Ga, ^86^Y, and ^177^Lu. Compared with FAPI-04, TEFAPI-06/07 showed improved tumor uptake and retention in a pancreatic cancer mice model. The mean %ID/g at 24- and 96-h post injection of [^177^Lu]Lu-TEFAPI-06 in tumor were 8.7 (± 0.7) and 7.3 (± 2.3), respectively, whereas the mean %ID/g to kidneys were 3.2 (± 0.8) and 2.7 (± 0.5), respectively. The doses to tumors of [^177^Lu]Lu-TEFAPI-06 and [^177^Lu]Lu-TEFAPI-07 were similar. However, doses to kidneys were higher with [^177^Lu]Lu-TEFAPI-07 (24 hours: 8.7 ± 2.3 %ID/g, 96 hours: 10.2 ± 3.3 %ID/g). Tumor growth inhibition was observed with [^177^Lu]Lu-TEFAPI-06 and [^177^Lu]Lu-TEFAPI-07 and was higher compared to [^177^Lu]Lu-FAPI-04 and a control group. No clinical trial of TEFAPI-06 or TEFAPI-07 is yet reported.

#### FAPI-C12 and FAPI-C16

Zhang et al. conjugated two fatty acids, lauric acid (C12) and palmitic acid (C16) to FAPI-04, to create two albumin-binding FAPI radiopharmaceuticals: FAPI-C12 and FAPI-C16, respectively [45]. Whole-body SPECT imaging of [^177^Lu]Lu-FAPI-C12, [^177^Lu]Lu-FAPI-C16, and [^177^Lu]Lu-FAPI-04 showed longer circulation time and significantly higher tumor uptake compared to FAPI-04. Moreover, [^177^Lu]Lu-FAPI-C16 had a higher tumor uptake at both 24 h (11.22 ± 1.18 %ID/g) and 72 h (6.50 ± 1.19 %ID/g) compared to [^177^Lu]Lu-FAPI-C12 (24 h, 7.54 ± 0.97 %ID/g; 72 h, 2.62 ± 0.65 %ID/g). The tumor uptake of [^177^Lu]Lu-FAPI-04 was much lower (1.24 ± 0.54 %ID/g at 24 h after injection). 29.6 MBq [^177^Lu]Lu-FAPI-C16 resulted in significant tumor volume inhibition in HT-1080-FAP tumor-bearing mice. The median survival with [^177^Lu]Lu-FAPI-C16 (28 days) was much longer compared with [^177^Lu]Lu-FAPI-C12 (12 days) or [^177^Lu]Lu-FAPI-04 (10 days) which was much longer than that of the [^177^Lu]Lu-FAPI-04-treated group of which the median survival was only 10 days.

#### FSDD

FSDD are FAP tracers with an albumin-binding moiety. In this study, FSDD_0_I, FSDD_1_I, and FSDD_3_I, were labeled with ^68^Ga and ^177^Lu and tested in human hepatocellular carcinoma patient-derived xenografts (HCC-PDXs) [46]. PET imaging showed that [^68^Ga]Ga-FSDD_0_I had a better blood retention and tumor uptake compared with [^68^Ga]Ga-FAPI-04, [^68^Ga]Ga-FSDD_1_I, and [^68^Ga]Ga-FSDD_3_I, and was therefore selected for further studying. For biodistribution studies, FSDD_0_I was labeled with ^177^Lu. At 1-h post injection, [^177^Lu]Lu-FSDD0I had high accumulated in the tumor (14.44 ± 1.174 %ID/g), bone (12.11 ± 2.83 %ID/g), and blood (10.30 ± 2.03 %ID/g). The authors postulate that the high bone uptake may be related to the physiological expression of FAP in murine osteoblasts and bone marrow stem cells. At the latest time point (48-h post injection), the ID%/g in tumor was ~ 5. The kidney uptake at 1- and 48-h post injection was ~ 5 ID%/g and ~ 1 ID%/g, respectively. Because of this, the authors will select FSDD01 for their following studies. Hitherto, no prospective study is registered.

## Discussion

This review provides an overview of the currently reported therapeutic tracers that target FAP for TRT. FAP is overexpressed by cancer-associated fibroblasts of several tumor types [47–49]. While the first outcomes of prospective trials of FAP TRT are still awaited, preclinical studies and selected case series showed encouraging data that FAP TRT may become a treatment option for different cancer types. Moreover, in current studies, FAP TRT seemed well tolerated, which corroborates with FDA-approved TRT in patients with neuro-endocrine tumors ([^177^Lu]Lu-DOTATE) and castration-resistant prostate cancer ([^177^Lu]Lu-PSMA).

With the recent success of PSMA ligands in prostate cancer (e.g., [^177^Lu]Lu-PSMA-617), many investigators have put their attention to the development of FAP tracers. To date, several FAP tracers have been reported with most data referring to four compounds: FAPI-04, FAPI-46, FAP-2286, and DATAGA.(SA.FAPI)_2_. Since *compassionate care* programs differ across the world, with some countries having a liberal policy, there is reported literature of more than 100 patients that have received TRT using FAP tracers. At present, the most promising clinical data of FAP TRT was reported in advanced sarcoma, breast, thyroid, and pancreatic cancer [30, 33]. In these end-stage patients, there is an unmet need for novel anti-cancer therapies. However, the present studies had a retrospective study design with potential selection bias to more fit patients. Moreover, most studies had suboptimal protocols (e.g., dosimetry from whole-body scans using minimal time points, non-randomized, no systematic reporting on AE or outcomes) with short follow-up which may have skewed the outcomes. Importantly, while it is anticipated that most of these malignancies are sensitive to radiation as external beam radiotherapy generally result in a response, it is unknown whether alfa or beta radiation will also result in tumoricidal effects. Therefore, the present reports only suggest that FAP TRT may have efficacy in advanced cancer patients, and results of the first prospective studies are warranted.

To date, the reported side effects show a favorable toxicity profile with limited and manageable high-grade adverse events. The most important reported adverse events are related to bone marrow toxicity (anemia, leukopenia, and reduced number of platelets). This is in concordance to known side effects of [^177^Lu]Lu-PSMA and [^177^Lu]Lu-DOTATATE. At present, it is not feasible to determine which FAP inhibitor has the best toxicity profile, because of the small cohorts and lack of prospective data with systematic reporting on AEs. Yet, the present real-life data suggest that toxicity is low which supports further studying.

Although studies mostly relied on whole-body scans with limited number scanning of time points, there is dosimetry data of FAP TRT for tumors and the organs at risk (e.g., bone marrow and kidneys). The absorbed dose to the tumor ranges from 0.62 ± 0.55 Gy/GBq, 2.81 ± 1.25 Gy/Gbq, 3.0 ± 2.7 Gy/GBq, and 6.70 (IQR: 3.40–49) Gy/GBq, for [^177^Lu]Lu-FAPI-04, [^90^Y]Y-FAPI-46, [^177^Lu]Lu-FAP-2286, and [^177^Lu]Lu-DOTAGA.(SA.FAPi)_2_, respectively. These dosimetry outcomes are in line with the tumor-absorbed doses of [^177^Lu]Lu-DOTATATE and [^177^Lu]Lu-PSMA-617 in neuro-endocrine tumors and prostate cancer, which are 4.4 (range: 0.1–32.0) and 3.25 ± 3.19 Gy/GBq, respectively [50–52]. The doses to the bone marrow and kidneys of the FAP tracers are also within range of [^177^Lu]Lu-DOTATATE and [^177^Lu]Lu-PSMA-617 [50–52]. This data suggests that sufficient radiation doses are absorbed by tumors using FAP TRT, and thus, responses can be anticipated.

The most important challenges of FAP TRT are related to the retention time of FAP tracers in tumor, which is pivotal to enable tumoricidal doses. At present, the best results are reported on [^177^Lu]Lu-FAP-2286, [^177^Lu]Lu-DOTAGA.(SA.FAPi)_2_, [^177^Lu]Lu-PNT6555, and the albumin-binding tracers (e.g., [^177^Lu]Lu-EB-FAP) showing uptake up to 168-h post injection. However, this also seemed to increase the dose to the organs at risk. Other investigators tried to overcome the issue of short tumor retention by labelling it (e.g., FAPI-46) with ^90^Y, which has a shorter T_1/2_ of 2.7 days vs 6.6 days of ^177^Lu. Therefore, the physical half-life of the isotope better matched the biological half-life of the tracer. Future studies will tell whether FAP tracers with prolonged retention time or FAP tracers with faster pharmacokinetics and shorter living radionuclides end up in better results. Moreover, we need to identify the malignancies that have good expression of FAP and are sensitive to alfa or beta radiation. Hence, most tumors show FAP expression in the TME, while some cancers actually express FAP on their cellular membrane (e.g., sarcoma, certain ovarian, and pancreatic cancers) [53]. Therefore, basket studies including different malignancies are awaited. On the other hand, this broad applicability is also its main opportunity with its potential use in a whole array of cancers. It is also postulated that FAP TRT is particularly useful in combination with other anti-cancer treatments. Here, FAP TRT could increase the permeability of the TME resulting in higher efficacy of concomitant chemo- and immunotherapies. All in all, we now await the first prospective phase I/II data of FAP TRT which are anticipated in 2023.

## Conclusion

The long circulation time of FAP-targeting antibodies and poor tumor retention time of FAP-targeting small molecules used to be a matter of concern. However, several teams have developed methods to overcome this limitation by labeling small molecules that target FAP to radionuclides with shorter half-lives. Moreover, there is incremental reporting of novel tracers that show tumor uptake up to 168-h post injection. To date, data was reported on more than 100 patients that were treated with different FAP-targeted radionuclide therapies such as [^177^Lu]Lu-FAPI-04, [^90^Y]Y-FAPI-46, [^177^Lu]Lu-FAP-2286, [^177^Lu]Lu-DOTA.SA.FAPI, and [^177^Lu]Lu-DOTAGA.(SA.FAPi)_2_. In these case series, FAP-targeted radionuclide therapy has resulted in objective responses in difficult to treat end-stage cancer patients with manageable adverse events. Although no prospective study data is yet available, the early data encourages further studying. For now, we await the first prospective phase I/II data of FAP TRT.

## Supplementary Information

Below is the link to the electronic supplementary material.

Supplementary file1 (DOCX 13 kb)

## Data Availability

Data sharing not applicable to this article as no datasets were generated or analyzed during the current study.
