# Supplementary material for: Fibroblast activation protein-targeted radionuclide therapy: background, opportunities, and challenges of first (pre)clinical studies
Source: Eur J Nucl Med Mol Imaging. 2023 Feb 23;50(7):1906–18. doi: 10.1007/s00259-023-06144-0 (PMC10199876; doi:10.1007/s00259-023-06144-0)
Supplement: Supplementary file 1 — Supplementary file1 (DOCX 13 kb) [file 259_2023_6144_MOESM1_ESM.docx]

**SUPPLEMENTARY MATERIAL**

Supplementary table 1: search strategy PubMed

Exploratory searches PubMed (22-07-2022) used for inclusion (as described in methods)

| **Type of therapy**  Search string | **Hits** |
| --- | --- |
| **FAPI-04**  ((((((((((cancer associated fibroblast) AND (fibroblast activation protein)) OR (FAP)) OR (FAPI)) AND (radionuclide)) AND (radioligand)) AND (radiotherapy)) OR (radio therapy)) AND (theranostics)) AND (cancer therapy)) OR (FAPI-04) | **444** |
| **FAPI-46**  ((((((((((cancer associated fibroblast) AND (fibroblast activation protein)) OR (FAP)) OR (FAPI)) AND (radionuclide)) AND (radioligand)) AND (radiotherapy)) OR (radio therapy)) AND (theranostics)) AND (cancer therapy)) OR (FAPI-46) | **215** |
| **FAP-2286**  ((((((((((cancer associated fibroblast) AND (fibroblast activation protein)) OR (FAP)) OR (FAPI)) AND (radionuclide)) AND (radioligand)) AND (radiotherapy)) OR (radio therapy)) AND (theranostics)) AND (FAP ligands)) OR (FAPI-04) | **200** |
| **ND-bis-FAPI**  ((((((((((cancer associated fibroblast) AND (fibroblast activation protein)) OR (FAP)) OR (FAPI)) AND (radionuclide)) AND (radioligand)) AND (radiotherapy)) OR (radio therapy)) AND (theranostics)) AND (FAP ligands)) OR (ND-bisFAPI) | **5** |
| **PNT6555**  ((((((((((cancer associated fibroblast) AND (fibroblast activation protein)) OR (FAP)) OR (FAPI)) AND (radionuclide)) AND (radioligand)) AND (radiotherapy)) OR (radio therapy)) AND (theranostics)) AND (FAP ligands)) OR (PNT6555) | **4** |
| **DOTA-FAPI**  ((((((((((cancer associated fibroblast) AND (fibroblast activation protein)) OR (FAP)) OR (FAPI)) AND (radionuclide)) AND (radioligand)) AND (radiotherapy)) OR (radio therapy)) AND (theranostics)) AND (FAP ligands)) OR (DOTA-FAPI) | **16** |
